# Age-related cognitive complaints and emotional difficulties associated with symptoms of ADHD: a study of gender differences

**DOI:** 10.3389/fgwh.2025.1607464

**Published:** 2025-10-07

**Authors:** Brandy L. Callahan, Emma A. Climie, Hawra Al-Khaz’Aly, Kate T. McKay

**Affiliations:** 1 Department of Psychology, University of Calgary, Calgary, AL, Canada; 2 Hotchkiss Brain Institute, Calgary, AL, Canada; 3 Werklund School of Education, University of Calgary, Calgary, AL, Canada

**Keywords:** ADHD, aging, cognition, emotion, emotional dysregulation, neurodiversity, gender differences, subjective cognitive impairment

## Abstract

**Background:**

Recent research suggests that attention-deficit/hyperactivity disorder (ADHD) is a risk factor for suboptimal cognitive and emotional aging. Due to menopause, women may be more vulnerable to these outcomes than men. This study quantifies age-related changes in the association between self-reported ADHD symptoms and cognitive and emotional complaints, comparing men and women.

**Methods:**

Participants were 118 community adults aged 19–79 years (78.0% women). Most had a self-reported ADHD diagnosis (71.2%) or clinically significant ADHD symptoms (78.0%). All completed the self-report Connors Adult ADHD Rating Scale, the Cognitive Failures Questionnaire, the Barkley Deficits in Executive Functioning Scales and the Difficulties in Emotion Regulation Scale. Gender-stratified general linear models predicted cognitive and emotional difficulties from ADHD symptoms, testing age as a moderator.

**Results:**

ADHD symptoms showed moderate to strong correlations with all cognitive (.39 < *r* < .68) and emotional outcomes (.21 < *r* < .64). In men, the association between ADHD symptoms and cognitive (*B* = −0.009, *p* = .021, *η*_p_^2^ = .23) and emotional impulsivity (*B* = −0.017, *p* = .012, *η*_p_^2^ = .28) was less pronounced in older than younger participants. Theses patterns were not observed in women. In older women, the association between ADHD symptoms and self-reported cognitive failures was slightly weaker than in younger women (*B* = −0.017, *p* = .030, *η*_p_^2^ = .05). Although this interaction was not statistically significant in men, the effect was of similar medium-sized magnitude (*η*_p_^2^ = .08). All associations survived adjustments for depression and anxiety symptoms.

**Conclusion:**

Some cognitive and emotional difficulties associated with ADHD symptoms were worse in younger than in older men, but age moderation was not observed in women. The cross-sectional design precludes any conclusions about causality, and it is possible that these results may be explained by greater self-disclosure in women than in men. Results are also interpreted cautiously in the context of relatively small sample size. Altogether, results support the need for a gender-specific lens when considering the lifespan impacts of ADHD symptoms and point to women as a potentially vulnerable segment of the ADHD population regarding cognitive and emotional aging.

## 1 Introduction

The first children to receive a diagnosis of attention-deficit/hyperactivity disorder (ADHD) when it was initially defined under the name ‘hyperkinetic reaction of childhood’ (1) are now entering their fifth and sixth decades of life. Concurrently, there has been improved recognition of ADHD across the lifespan in recent years, with significant increases in older adults receiving a diagnosis in the last decade (2). As a result, roughly one in six people currently living with ADHD worldwide is over the age of 50 (3, 4), with many more are aging into this demographic each year. The prevalence of ADHD is estimated to be around 5%–7% in childhood and 3%–5% in adulthood, although diagnostic rates in adults are complicated by heterogeneous symptom presentations (5) and difficulty establishing age of onset in older individuals (6). In general, symptoms of hyperactivity tend to be lower in adults than in children, while symptoms of inattention are common in adults (7).

Emerging research has begun to explore age-related outcomes in ADHD, and we are beginning to construct an understanding of how ADHD affects lifespan aging outcomes. Findings from these initial studies are generally bleak, with converging evidence from several large population studies pointing to an increased risk of suboptimal cognitive aging among older adults with ADHD relative to their same-aged peers without ADHD (8, 9). After controlling for potential confounding factors such as psychiatric and physical comorbidities, lifestyle, and socioeconomic factors, ADHD is associated with a roughly 3- to 4-fold increased hazard of all-cause dementia (10–12) and a 2.5-fold increased hazard of mild cognitive impairment [MCI; (10)]. MCI is often considered an early manifestation of dementia (13). An even earlier predictor of pathological cognitive aging, appearing several years before MCI, is subjective cognitive impairment [SCI; (14)]. SCI is defined as the personal perception of a reduction in cognitive function relative to past performance or to peers, in the absence of objective cognitive impairment (15). In other words, it refers to the experience of cognitively normal individuals who feel that their thinking abilities are impaired relative to others their age or have notably declined relative to previous levels. SCI is a strong independent predictor of future cognitive decline in many individuals (14), particularly women (16). Its association with ADHD has not yet been explored but could provide valuable information about cognitive aging in the context of ADHD.

Alongside potential cognitive challenges, adults with ADHD also appear to experience emotional difficulties in later life relative to their peers without ADHD (17). They experience more frequent rates of depression and anxiety (18, 19), emotional loneliness (20) and burnout (21). These emotional challenges may potentially result, in part, from deficits in emotional regulation [i.e., the ability to control one's emotional responses; (22)]. Emotion dysregulation is a well-established feature of ADHD (23, 24) and is linked to general psychological well-being (25). It is distinct from emotional manifestations of other psychiatric conditions that may co-occur alongside ADHD and directly impacts functional outcomes across several domains and quality of life (23, 24). It is necessary to understand how this facet of ADHD may be expressed differently across men and women of different ages to more completely describe the experience of aging with ADHD.

In the general population, there is a vast literature indicating that aging is associated with overall increases in positive affect (26). This is presumably due to improved age-related abilities in emotional regulation and increased motivation to persue pleasurable activities in daily life [(27); but see (28)], a phenomenon referred to as socioemotional selectivity. Socioemotional selectivity theory predicts that older adults are conscious of their limited time left to live and therefore regulate their emotional states to optimize well-being during this time (26). ADHD could subvert this phenomenon, conceivably because of ineffective control of attentional resources and/or as a result of time blindness (i.e., inaccurate estimation of temporal relationships), both of which are characteristic of ADHD (29, 30). To our knowledge, emotional processes in ADHD have only been explored past midlife in one study, which asked young and older adults with ADHD to self-rate their general psychological health using questions that encompassed emotional fluctuations and overreactions, among other things (31). Results showed that the broad psychological health construct was rated as better by older adults, suggesting that older adults with ADHD may experience fewer deficits in emotion regulation than younger adults. This finding would be consistent with general emotion regulation improvements predicted by socioemotional selectivity. This needs further empirical verification using more direct measures of emotional regulation ability.

There is reason to anticipate that women with ADHD may be disproportionately impacted by age-related changes relative to men due to mid-life hormonal changes that can have adverse impacts on both cognitive and emotional health. As women transition through menopause into later life, between 50%–75% will experience distressing symptoms due to hormonal fluctuations (32). Among these symptoms, cognitive changes are the most commonly reported concerns after hot flashes (33) and involve primarily perceived changes in attention, episodic and/or working memory, and concentration (34). Interestingly, self-reported SCI may be more reliably associated with menopausal changes than objective cognitive test scores (33, 34), potentially explicable by comorbid depressive or vasomotor symptom severity (35, 36). In most women, these cognitive challenges are isolated to the perimenopausal period (32). However, there have been some reports of faster cognitive decline in women than men (37) as they enter their sixties and seventies, despite higher general cognitive abilities than same-aged men prior to menopause (37, 38). The perimenopausal period is also one during which women may experience increased difficulty regulating their emotions (22, 39) as a result of deficient estrogen, a principal regulatory hormone (40). Despite the emotion dysregulation being a prominent feature of ADHD (23), age-related changes emotional regulation in women have not been adequately explored.

The present study adopts a gender-specific lens to augment existing knowledge about age-related cognitive (9) and emotional outcomes (20) linked to ADHD symptoms in women and men.We quantify symptoms of inattention, hyperactivity and impulsivity using scales that have been validated for use in adults, and henceform refer to this symptom triad as “ADHD symptoms” for the purposes of this work. We intentionally adopt a dimensional, symptomatic perspective (as opposed to a diagnostic definition of ADHD), because inattention, hyperactivity and inattention may adversely impact functioning and well-being even when they are subclinical or undiagnosed (41, 42). There is also evidence that women experience a four-year delay in receiving an ADHD diagnosis relative to men (43) and consequently may experience impairing symptoms of inattention and hyperactivity despite not having a formal diagnosis. The objective of the study is to explore gender differences in the modifying effect of age on the relationship between self-reported ADHD symptoms and cognitive and emotional difficulties, in a sample of adults aged 18–80 years. Given the age-related hormonal changes experienced by women that are known to adversely impact cognitive and emotional health, it is expected that older women will experience worse ADHD-related outcomes than younger women, and than older and younger men.

We deliberately focus on participants’ subjective (i.e., self-reported) perceptions of their cognitive and emotional health because there is robust evidence that these measures may provide more accurate estimates of everyday abilities relative to objective (i.e., performance-based) measures among adults with ADHD (44). In this context, subjective measures have greater ecological validity because they inquire about performance in natural, real-world situations, as opposed to the structured, distraction-free laboratory environment. In addition, they capture behaviors over a much broader time frame (usually weeks or months) whereas laboratory tests capture performance at single point in time that may not be reflective of the participant's standard ability. Moreover, rating scales provide estimates of valuable components associated with the meta-construct of executive functioning that are not captured by performance-based measures (e.g., self-motivation, self-regulation). Also relevant to the central objective of this study is that subjective perceptions of cognition are independently associated with cognitive aging outcomes: regardless of actual cognitive performance, one's subjective impression of impairment or decline is a strong predictor of later dementia (15). Because our study is predicated on the need to elucidate potential risk factors for unsuccessful aging in ADHD, subjective complaints are therefore highly relevant to explore.

## 2 Materials and methods

### 2.1 Participants

Participants in this study were recruited from the Calgary community in Alberta, Canada as part of a larger longitudinal cohort exploring predictors of psychosocial outcomes related to symptoms of ADHD. Eligibility criteria for this larger study included sufficient fluency in English to complete all questionnaires, age 18 or older, and normal or corrected-to-normal hearing and vision. Stroke or dementia were exclusionary; MCI was not. Participants were not required to have ADHD to participate, but all were asked upon enrollment whether they had a confirmed or suspected ADHD diagnosis (yes or no). To receive a formal diagnosis of ADHD in Canada, thorough assessment by a healthcare practitioner is required (only family doctors, pediatricians, psychiatrists, nurse practitioners, and psychologists can provide this service). Practitioners normally apply the Canadian ADHD Practice Guidelines (45), which indicate that the assessing clinician must be licensed and adequately trained in the application of Diagnostic and Statistical Manual Fifth Edition [DSM-5; (46)] diagnostic criteria for ADHD. The patient must meet DSM−5 criteria for ADHD, which require at least five symptoms of inattention or impulsivity/hyperactivity that are longstanding since childhood, cause impairment in multiple domains of functioning, and are not better explained by an alternate or circumstantial condition. The Canadian guidelines further indicate that the clinician must assess the frequency and severity of symptoms and impairment using a comprehensive clinical interview (which must include a complete childhood developmental history and review of past medical records) in combination with valid, reliable and sensitive rating scales and incorporating corroborating reports from knowledgeable informants. The assessment must also reflect an understanding of multi-systemic issues that may confound or complicate the ADHD diagnosis. ADHD diagnosis was not used as a variable of interest in this study's statistical analyses, but this information was collected simply to characterize the sample.

Regardless of their diagnostic status, all participants in this completed ADHD symptom measures, described below, to estimate symptom presence and severity. All participants gave written informed consent to participate, and study procedures were carried out in accordance with the Declaration of Helsinki and were approved by the University of Calgary Conjoint Faculties Research Ethics Board (CFREB#20-1103).

### 2.2 Measures

Participants received an email link to complete an online questionnaire, programmed in the Qualtrics survey environment, in which they were asked to provide basic sociodemographic data (age, gender, education level, and ethnic background) and to complete various cognitive and behavioral measures, described below.

#### 2.2.1 ADHD symptom severity

The Adult ADHD Self-Report Scale [ASRS; (47)] was used to capture ADHD symptom severity. This tool consists of six items measuring the frequency of inattention (four items) and hyperactivity (two items) over the prior six months, from 0 (never) to 4 (very often). It is among the most commonly used screening tools for ADHD (48), and has the advantage of being brief and free of cost. Responses can be tallied in several ways; the present study summed all scores ranging from 0 to 24, and scores ≥14 were interpreted to reflect clinically significant ADHD symptoms. This method of scoring the ASRS is associated with good sensitivity (65%) and excellent specificity (94%), out-performing other scoring methods in terms of its ability to capture symptom severity better and its concordance with clinical diagnoses (49). This method also shows strong test-retest reliability [*r* = .58–.77; (49)]. We note that the ASRS is intended only as an indication of presence and severity of inattention and hyperactivity, and cannot be used diagnostically.

The Self-Report Short Form of the Conners Adult ADHD Rating Scale [CAARS; (50)] was used to capture symptom severity across hyperactive/impulsive and inattentive subscales separately. This 26-item self-report scale was derived from a longer, 66-item version of the CAARS and includes only items that best discriminated ADHD (51). The short version measures the frequency of inattentive, hyperactive, and impulsive symptoms on a scale from 0 (never) to 3 (very frequent), with a total score ranging from 0 to 78. For this study, raw scores were transformed to age- and gender-adjusted *T* scores based on published normative data (50), where *T* > 65 corresponds to symptom severity falling 1.5 standard deviations (*SD*) above average. Similar to the ASRS, the CAARS cannot be used diagnostically.

#### 2.2.2 Symptoms of depression and anxiety

Given that ADHD symptoms are highly correlated with other mental health comorbidities (52–54), symptoms of depression and anxiety over the previous two weeks were ascertained using the Patient Health Questionnaire [PHQ-9; (55)] and the Generalized Anxiety Disorder 7-item scale [GAD-7 (56);], respectively. These are brief, self-reported questionnaires measuring the frequency of symptoms on a scale from 0 (not at all) to 3 (nearly every day), with higher scores indicating more severe symptoms. The PHQ-9 has excellent sensitivity (88%) and specificity (88%) with good internal consistency [*α* = .83; (57)]. The PHQ-9 also has moderate to strong convergent validity as per correlation with different measures of depression [*r* = .48–.68, *p* < .001 (57)]. Similarly, the GAD-7 has excellent sensitivity (89%), specificity (82%), test-retest reliability (*r* = .83) and good criterion, construct, and convergent validity [*r* = .72-–.74, *p* < .05; (56)]. These scales were selected for use in the present study because they are brief and freely available, are widely used in clinical and research protocols, and are generally invariant across diverse groups (58).

#### 2.2.3 Cognitive complaints

Cognitive complaints were quantified using Barkley Deficits in Executive Functioning Scale [BDEFS; (59)] and the the Cognitive Failures Questionnaire [CFQ; (60)]. The BDEFS is a 20-item self-report measure which was used to quantify the extent to which participants experience daily difficulties with time management, everyday organization, self-restraint, ability to self-motivate and regulate emotions. In each of these domains, participants are asked to rate their level of difficulty ranging from 1 (rarely or never) to 4 (very often). Raw scores were transformed to age- and gender-adjusted *T* scores based on published normative data (59), where *T* > 65 corresponds to symptom severity falling 1.5 standard deviations (*SD*) above average. The BDEFS was chosen for its excellent internal consistency (*α* > .90) and good reliability [*r* = .62-.80, *p* < .001; (61)], and studies have supported its validity for evaluating executive dysfunction in adults (59, 61)

The CFQ is a 25-item questionnaire asking participants to estimate the frequency of different slips in memory, attention, language, and impulse control they may have encountered over the previous six months, on a scale from 0 (never) to 4 (very often). All items are summed to produce a total score ranging from 0 to 100, with higher scores indicating more perceived cognitive difficulties. This measure was selected as a complement to the BDEFS, which focuses exclusively on executive functioning. The CFQ demonstrates excellent internal consistency (*α* > .88) and reliability [*r* > .71; (62, 63)], and its factor structure and measurement properties are invariant across the adult lifespan (64).

#### 2.2.4 Emotional dysregulation

The Difficulties in Emotion Regulation Scale [DERS; (65)] was used as a measure of emotional dysregulation. This 36-item questionnaire yields measures of nonacceptance of emotional responses (e.g., feeling ashamed or guilty about getting upset), difficulty engaging in goal-directed behavior when upset (i.e., being unable to redirect one's thoughts or emotions), emotional impulse control difficulties (i.e., a feeling of lack of control over one's emotions), difficulties with emotional awareness (i.e., lacking insight into one's emotional reactions), difficulties using emotion regulation strategies (e.g., believing that bad feelings will never go away), and lack of emotional clarity (i.e., being able to name and understand felt emotions). On each scale, higher scores indicate worse emotion dysregulation. The DERS has excellent internal consistency across different racial groups and genders [*α* > .92; (66)], and moderate construct and convergent validity [*r* > .57; (66)].

### 2.3 Statistical analyses

All statistical analyses were conducted in SPSS v.26 for Windows. Participants’ sociodemographic characteristics were summarized using descriptive statistics. Participant age, ADHD symptoms and cognitive and emotional complaints were compared between men and women using Student's *t* tests. Confirmed ADHD diagnosis by a healthcare professional (% yes), Education (levels) and ethnicity (% White) were compared across men and women using chi-square. Two multivariate general linear models were built with ADHD symptoms (CAARS ADHD Index) as continuous covariates (i.e., independent variables) and cognitive (CFQ, BDEFS) and emotional difficulties (DERS) as the dependent variables; both models included an age-by-ADHD symptom interaction term to test the modifying effect of age on ADHD-related outcomes. The model can be formulated as:

$$\begin{array}{c} Y\\ \left(n\times m\right) \end{array}=\begin{array}{c} X\\ \left(n\times k+1\right) \end{array}\begin{array}{c} B\\ \left(k+1\times m\right) \end{array}+\begin{array}{c} E\\ \left(n\times m\right) \end{array}$$

where *Y* is a matrix of *n* observations on *m* outcomes (in this case, CFQ and BDEFS scores in one model, and DERS scores in a second model); *X* is a model matrix for *k* predictors (in this case, age, CAARS ADHD Index, and the age-by-ADHD Index interaction in both models) plus a regression constant; B is a matrix of the regression coefficients associated with the predictors and the constant; and E is a matrix of errors (67). The models were gender-stratified to compare potential age effects across men and women. Adjusted models controlled for symptoms of depression and anxiety. All model residuals were normally distributed. Normality of residuals was ascertained by visually inspecting the standardized residuals of the predicted values. Homogeneity of variances across men and women could not be calculated directly because the models were gender-stratified (i.e., gender was a not a between-subjects factor) but the spread of dependent variables in both groups was found to be generally comparable by visual inspection of the boxplots.

## 3 Results

Sample characteristics are summarized in Table 1. Participants (*N* = 118) were aged 19–79 years with a mean (*M*) age of 41.5 years [standard deviation (*SD*) = 17.3]. Roughly three-quarters of the sample (78.0%) identified as women, and most (55.9%) had university-level education. A formal diagnosis of ADHD by a healthcare practitioner was self-reported by 84 individuals (71.2%) and 14 additional people (11.9%) strongly suspected they had ADHD but had never been diagnosed. ASRS scores ranged from 4 to 24 (*M* = 16.2, *SD* = 4.15), and 92 people (78.0%) fell above the ASRS cut-off indicating clinically significant ADHD symptoms.

**Table 1:** Sample characteristics.

Characteristics	Men (*n* = 26)		Women (*n* = 92)		Test statistic	*p* value	Effect size
	Range or *N*	Mean (*SD*) or %	Range or *N*	Mean (*SD*) or %			
Age	21–79	51.12 (18.86)	19–74	39.84 (15.74)	*t* = 3.085	.003	*d* = .685
Confirmed ADHD diagnosis (Yes)	14	53.8%	70	76.1%	*χ^2^* = 4.889	.027	*φ* = .204
ASRS total score	4–24	15.12 (4.64)	5–24	16.52 (3.97)	*t* = −1.535	.127	*d* = .341
Education					*χ^2^* = 3.492	.625	*φ* = .172
Completed middle school	1	3.8%	1	1.1%			
Completed high school	5	19.2%	22	23.9%			
Completed trade school	7	26.9%	16	17.4%			
Completed a Bachelor's degree	10	38.5%	32	34.8%			
Completed a Master's degree	3	11.5%	20	21.7%			
Completed a PhD or MD	0	0.0%	1	1.1%			
Ethnicity							
White	19	73.1%	79	85.9%	*χ^2^* = 2.357	.125	*φ* = .141
South Asian	3	11.5%	2	2.2%			
Southeast Asian	1	3.8%	4	4.3%			
East Asian	1	3.8%	3	3.3%			
Latin, Central or South American	1	3.8%	2	2.2%			
Black	0	0.0%	2	2.2%			
Middle Eastern	0	0.0%	1	1.1%			
Indigenous	0	0.0%	1	1.1%			
Other or Prefer not to say	1	3.8%	1	1.1%			
CAARS *T* scores							
Inattentive subscale	40–84	63.77 (10.80)	36–90	69.57 (11.30)	*t* = −2.331	.021	*d* = .518
Hyperactive subscale	40–77	59.35 (10.37)	38–74	60.95 (8.68)	*t* = −0.794	.429	*d* = .176
Impulsive subscale	40–76	53.85 (9.89)	37–85	57.55 (9.90)	*t* = −1.687	.094	*d* = .375
ADHD Index	39–80	63.81 (10.10)	45–87	66.64 (9.42)	*t* = −1.333	.185	*d* = .296

ADHD, attention-deficit/hyperactivity disorder; CAARS, conners adult ADHD rating scale; *SD*, standard deviation.

### 3.1 Correlations between variables of interest

Table 2 shows a correlation matrix of age and all variables of interest. Correlation coefficients *r* < .3, reflect weak correlations, those.3 < *r* < .7 reflect moderate correlations, and those *r* > .7 are considered strong. In the overall sample, the ADHD Index was significantly correlated with all cognitive (.39 < *r* < .68) and emotional outcomes (.21 < *r* < .64). In general, correlations were stronger in men than in women. Age, in particular, was moderately negatively correlated with multiple measures of cognitive and emotional complaints in men, but only weak or non-significant correlations for women.

**Table 2:** Correlation matrix summarizing Pearson correlations between study variables in the overall sample, and stratified by gender.

Overall sample (*N* = 118)																		
Variables	1.	2.	3.	4.	5.	6.	7.	8.	9.	10.	11.	12.	13.	14.	15.	16.	17.	18.
Age	–																	
CAARS subscales																		
Inattention	−0.138	–																
Hyperactivity	-.273**	.305**	–															
Impulsivity	−0.049	.422**	.290**	–														
ADHD Index	−0.04	.700**	.502**	.696**	–													
Cognitive complaints																		
Total CFQ	−.298**	.654**	.429**	.533**	.677**	–												
BDEFS Total	−0.103	.611**	.320**	.434**	.640**	.659**	–											
Time Mgmt	−.243**	.689**	.215*	.340**	.572**	.601**	.728**	–										
Organization	−0.038	.321**	.061	.314**	.398**	.463**	.599**	.455**	–									
Self-Restraint	−0.177	.381**	.371**	.623**	.608**	.595**	.564**	.380**	.361**	–								
Self-Motivation	−.309**	.525**	.108	.355**	.425**	.429**	.497**	.591**	.372**	.366**	–							
Self-Regulation	−.189*	.324**	.372**	.532**	.515**	.548**	.562**	.374**	.428**	.506**	.316**	–						
Emotional complaints																		
DERS Total	−0.178	.376**	.337**	.535**	.624**	.562**	.471**	.394**	.352**	.541**	.361**	.696**	–					
Non-Accept	−0.076	.256**	.133	.313**	.397**	.309**	.306**	.279**	.236*	.328**	.273**	.495**	.808**	–				
Goals	−.211*	.523**	.375**	.470**	.590**	.604**	.488**	.449**	.336**	.407**	.416**	.464**	.697**	.358**	–			
Impulse	−0.158	.322**	.317**	.681**	.637**	.552**	.430**	.302**	.340**	.658**	.279**	.616**	.842**	.542**	.609**	–		
Aware	0.124	.091	.089	.073	.213*	.102	.172	.132	.117	.197*	.078	.190*	.359**	.281**	.224*	.281**	–	
Strategies	−0.127	.297**	.339**	.440**	.552**	.477**	.407**	.364**	.309**	.444**	.290**	.692**	.912**	.643**	.611**	.722**	.342**	–
Clarity	−0.115	.231*	.225*	.277**	.396**	.363**	.341**	.279**	.220*	.383**	.300**	.428**	.600**	.383**	.375**	.475**	.668**	.534**

Women (*n* = 92)																		
Variables	1.	2.	3.	4.	5.	6.	7.	8.	9.	1.	11.	12.	13.	14.	15.	16.	17.	18.
Age	–																	
CAARS subscales																		
Inattention	−0.077	–																
Hyperactivity	−0.200	.316**	–															
Impulsivity	0.063	.414**	.316**	–														
ADHD Index	0.056	.711**	.505**	.703**	–													
Cognitive complaints																		
Total CFQ	−.227*	.657**	.444**	.482**	.635**	–												
BDEFS Total	0.008	.650**	.317**	.436**	.621**	.633**	–											
Time Mgmt	−0.099	.740**	.167	.293**	.548**	.562**	.712**	–										
Organization	0.003	.297**	.077	.276**	.366**	.449**	.614**	.417**	–									
Self-Restraint	−0.102	.378**	.345**	.653**	.604**	.594**	.594**	.361**	.380**	–								
Self-Motivation	−.245*	.576**	.097	.390**	.464**	.459**	.518**	.570**	.354**	.440**	–							
Self-Regulation	−0.056	.347**	.339**	.553**	.521**	.514**	.575**	.289**	.436**	.545**	.313**	–						
Emotional complaints																		
DERS Total	−0.095	.334**	.309**	.523**	.574**	.504**	.417**	.309**	.326**	.533**	.364**	.745**	–					
Non-Accept	−0.049	.218*	.099	.316**	.392**	.311**	.283**	.249*	.236*	.355**	.299**	.560**	.845**	–				
Goals	−0.165	.525**	.413**	.509**	.583**	.599**	.488**	.386**	.316**	.461**	.419**	.549**	.699**	.378**	–			
Impulse	−0.089	.281**	.288**	.692**	.600**	.497**	.418**	.242*	.317**	.658**	.320**	.670**	.842**	.594**	.627**	–		
Aware	0.203	−.002	.047	.097	.179	.106	.159	.042	.118	.149	.055	.255*	.369**	.328**	.170	.279**	–	
Strategies	−0.038	.263*	.307**	.433**	.487**	.400**	.340**	.260*	.277*	.419**	.268*	.725**	.914**	.704**	.623**	.699**	.329**	–
Clarity	0.012	.193	.186	.238*	.357**	.330**	.267*	.192	.206	.301**	.295**	.414**	.609**	.430**	.382**	.470**	.687**	.529**
Men (*n* = 26)																		
Variables	1.	2.	3.	4.	5.	6.	7.	8.	9.	1.	11.	12.	13.	14.	15.	16.	17.	18.
1. Age	–																	
CAARS subscales																		
2. Inattention	−0.113	–																
3. Hyperactivity	−.427*	.240	–															
4. Impulsivity	−0.218	.364	.186	–														
5. ADHD Index	−0.185	.641**	.481*	.649**	–													
Cognitive complaints																		
6. Total CFQ	−0.329	.572**	.374	.636**	.786**	–												
7. BDEFS Total	−0.327	.527**	.320	.431*	.711**	.750**	–											
8. Time Mgmt	−.554**	.493*	.323	.445*	.620**	.685**	.790**	–										
9. Organization	0.003	.312	−.029	.380	.460*	.437*	.571**	.538**	–									
10. Self-Restraint	−.404*	.401*	.452*	.526**	.627**	.637**	.503*	.443*	.285	–								
11. Self-Motivation	−.533**	.326	.14	.209	.262	.333	.449*	.671**	.438*	.060	–							
12. Self-Regulation	−.489*	.173	.452*	.422*	.467*	.624**	.535**	.615**	.360	.364	.323	–						
Emotional complaints																		
13. DERS Total	−.441*	.545**	.427*	.604**	.814**	.801**	.671**	.697**	.445*	.571**	.349	.536**	–					
14. Non-Accept	−0.243	.487*	.264	.387	.463*	.395	.417*	.434*	.270	.227	.167	.306	.667**	–				
15. Goals	−0.237	.465*	.254	.259	.585**	.582**	.494*	.615**	.364	.209	.415*	.167	.702**	.324	–			
16. Impulse	−0.299	.435*	.390	.654**	.744**	.720**	.469*	.467*	.398	.657**	.129	.443*	.849**	.372	.540**	–		
17. Aware	−0.210	.540**	.239	.093	.392	.202	.249	.503*	.168	.399	.175	.034	.349	.079	.483*	.331	–	
18. Strategies	−.420*	.456*	.437*	.530**	.783**	.785**	.610**	.713**	.430*	.537**	.374	.615**	.916**	.422*	.601**	.808**	.389	–
19. Clarity	−.582**	.405*	.372	.474*	.564**	.529**	.622**	.634**	.278	.752**	.324	.500*	.559**	.163	.359	.502*	.624**	.572**

* Correlation is significant at *p* < .05. **Correlation is significant at *p* < .01. ADHD, attention-deficit/hyperactivity disorder;Aware, difficulties with emotional awareness; CAARS, conner's adult ADHD rating scale; CFQ, cognitive failures questionnaire; Clarity, lack of emotional clarity; BDEFS, barkley deficits in executive functioning scale; DERS, difficulties in emotion regulation scale; Goals, difficulty engaging in goal-directed behavior when upset; Impulse, emotional impulse control difficulties; Non-Accept, nonacceptance of emotional responses; Strategies, difficulties using emotion regulation strategies; Time Mgmt, time management.

### 3.2 Gender differences in ADHD symptoms and subjective difficulties

Table 3 summarizes ADHD symptoms and cognitive and emotional complaints in men and women. Effect sizes are reported as *η*_p_^2^ where values of.01,.06 and.14 indicate weak, moderate and strong effects, respectively. Generally, men and women reported similar levels of ADHD symptom severity, with the exception of inattentive ADHD symptoms which were greater in women (*M* = 69.57, *SD* = 11.30) than in men (*M* = 63.77, *SD* = 10.80, *p* = .021, *d* = 0.52), and overall cognitive failures which were also greater in women (*M* = 60.35, *SD* = 15.76) than in men (*M* = 51.65, *SD* = 17.24, *p* = .017, *d* = 0.54).

**Table 3:** Gender differences in ADHD symptoms and emotional and cognitive outcomes.

Scores	Women (*n* = 92)	Men (*n* = 26)	*t*	*p*	Cohen's *d*
	Mean (*SD*)	Mean (*SD*)			
CAARS subscales					
Inattention *T* score	69.57 (11.30)	63.77 (10.80)	−2.331	.021	0.518
Hyperactivity *T* score	60.95 (8.68)	59.35 (10.37)	−0.794	.429	0.176
Impulsivity *T* score	57.55 (9.90)	53.85 (9.89)	−1.687	.094	0.375
ADHD Index *T* score	66.64 (9.42)	63.81 (10.10)	−1.333	.185	0.296
Cognitive difficulties					
Total CFQ score	60.35 (15.76)	51.65 (17.24)	−2.432	.017	0.540
Time management	12.90 (2.86)	12.04 (3.12)	−1.334	.185	0.296
Organization	9.73 (3.34)	8.54 (3.33)	−1.607	.111	0.357
Self-restraint	8.85 (3.03)	8.62 (2.97)	−0.347	.730	0.077
Self-motivation	8.41 (3.16)	8.16 (3.08)	−0.357	.722	0.081
Self-regulation	7.48 (2.51)	6.81 (2.59)	−1.195	.235	0.265
Total BDEFS *T* score^a^	59.69	56.46	0.426	.670	0.039
Emotional difficulties^b^					
Nonacceptance	15.93 (6.88)	16.63 (6.21)	0.447	.656	0.103
Goal-directed behavior	18.96 (4.56)	17.54 (4.76)	−1.336	.184	0.309
Impulse control	14.74 (5.61)	13.75 (5.97)	−0.754	.453	0.174
Emotional awareness	16.62 (5.34)	17.88 (5.36)	1.013	.313	0.234
Regulation strategies	20.92 (7.30)	21.17 (7.86)	0.145	.885	0.034
Emotional clarity	12.72 (4.61)	12.50 (3.88)	−0.211	.833	0.049
DERS total	68.24 (20.76)	66.38 (19.46)	−0.393	.695	0.091

a BDEFS *T* scores were not normally distributed, therefore men and women were compared using Mann–Whitney *U*; results reported under ‘Mean (*SD*)’ refer to mean rank, the value reported under ‘*t*’ refers to the standardized Mann–Whitney U test statistic, and the result reported under ‘Cohen's d’ refers to *r* where 0.039 represents a very small effect.

b DERS data were missing for two men and seven women. Effect sizes are shown as Cohen's *d* where 0.2 indicates a small effect, 0.5 indicates a medium effect and 0.8 indicates a large effect. ADHD, attention-deficit/hyperactivity disorder; CFQ, cognitive failures questionnaire; BDEFS, barkley deficits in executive functioning scale; DERS, difficulties in emotion regulation scale; *SD*, standard deviation.

### 3.3 Gender-stratified associations between ADHD symptoms, age, and cognitive complaints

**Figure 1:**
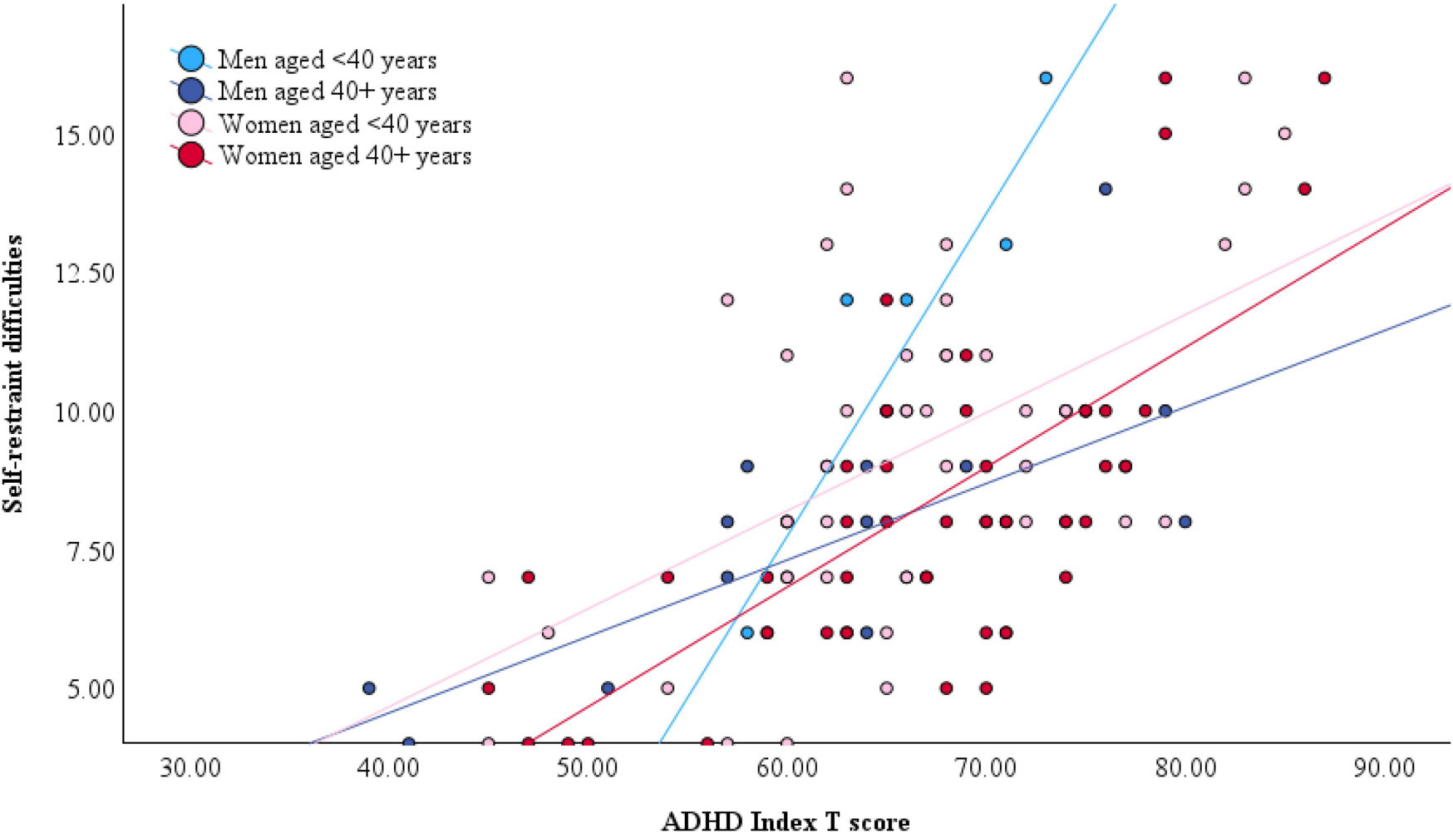
Gender-stratified relationship between self-restraint difficulties, ADHD symptom severity, and age. Note that age was dichotomized by median split for illustrative purposes only. All models included age as a continuous predictor.

**Figure 2:**
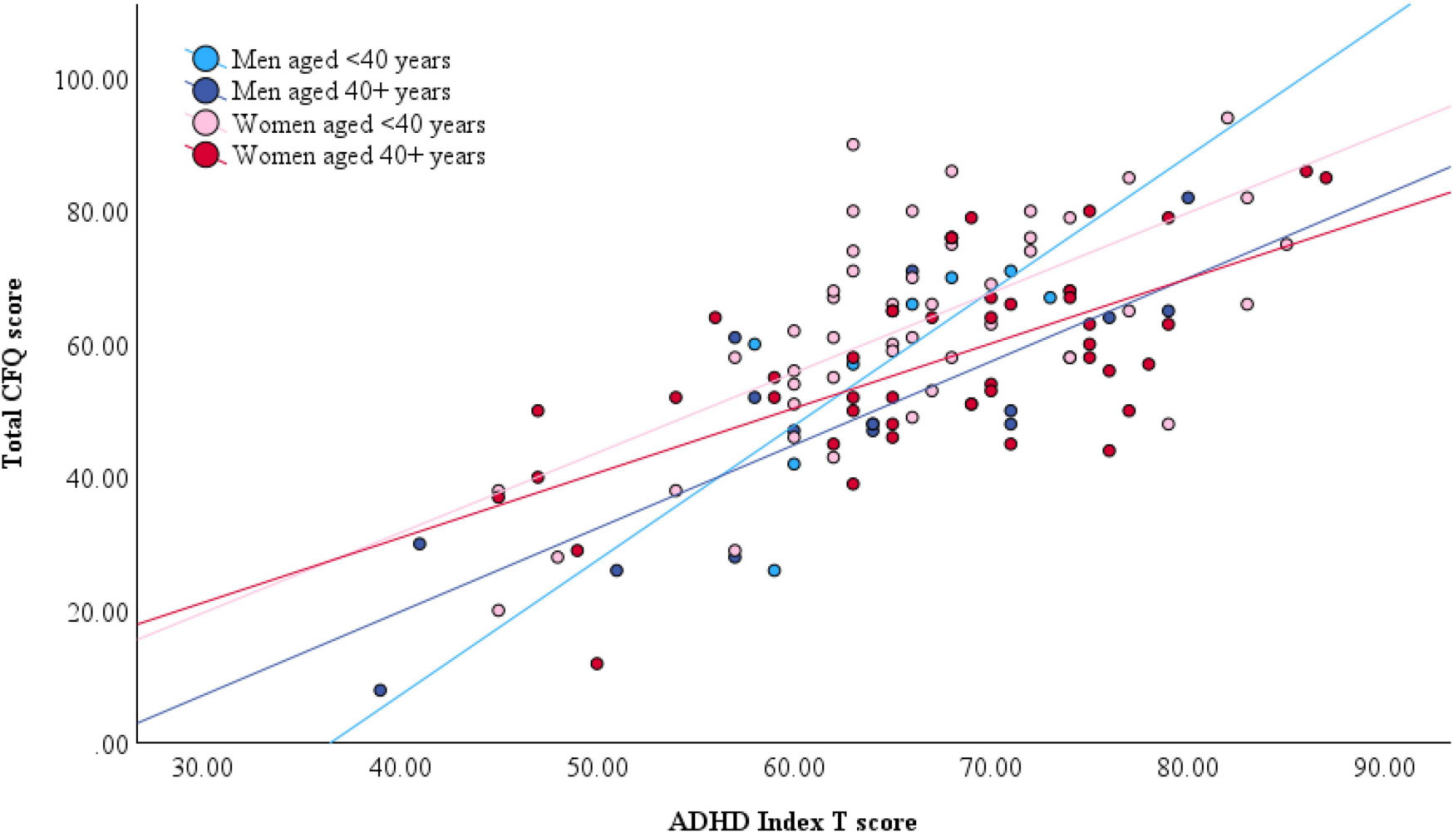
Gender-stratified relationship between total CFQ score, ADHD symptom severity, and age. Note that age was dichotomized by median split for illustrative purposes only. All models included age as a continuous predictor.

In men, age significantly moderated the relationship between ADHD symptoms and BDEFS self-restraint difficulties, whereby the association was stronger in younger than in older men (*B* = −0.009, *p* = .021, *η*_p_^2^ = .23). Age was not a significant moderator of this association in women (*B* = 0.001, *p* = .415, *η*_p_^2^ < .01) (Figure 1). In women, age only moderated the relationship between ADHD symptom severity and total cognitive failures, which was stronger in younger than in older women (*B* = −0.017, *p* = .030, *η*_p_^2^ = .05). Although this interaction was not statistically significant in men, the moderating effect was of similar magnitude (*B* = −0.025, *p* = .197, *η*_p_^2^ = .08) (Figure 2) and confidence intervals overlapped with those of women (Table 4). All associations survived adjustments for depression and anxiety symptoms and the magnitude of the effects remained similar. None of the other cognitive outcomes evidenced a moderating effect of age. Adjusted model parameters are summarized in Table 4.

### 3.4 Gender-stratified associations between ADHD symptoms, age, and emotional difficulties

**Figure 3:**
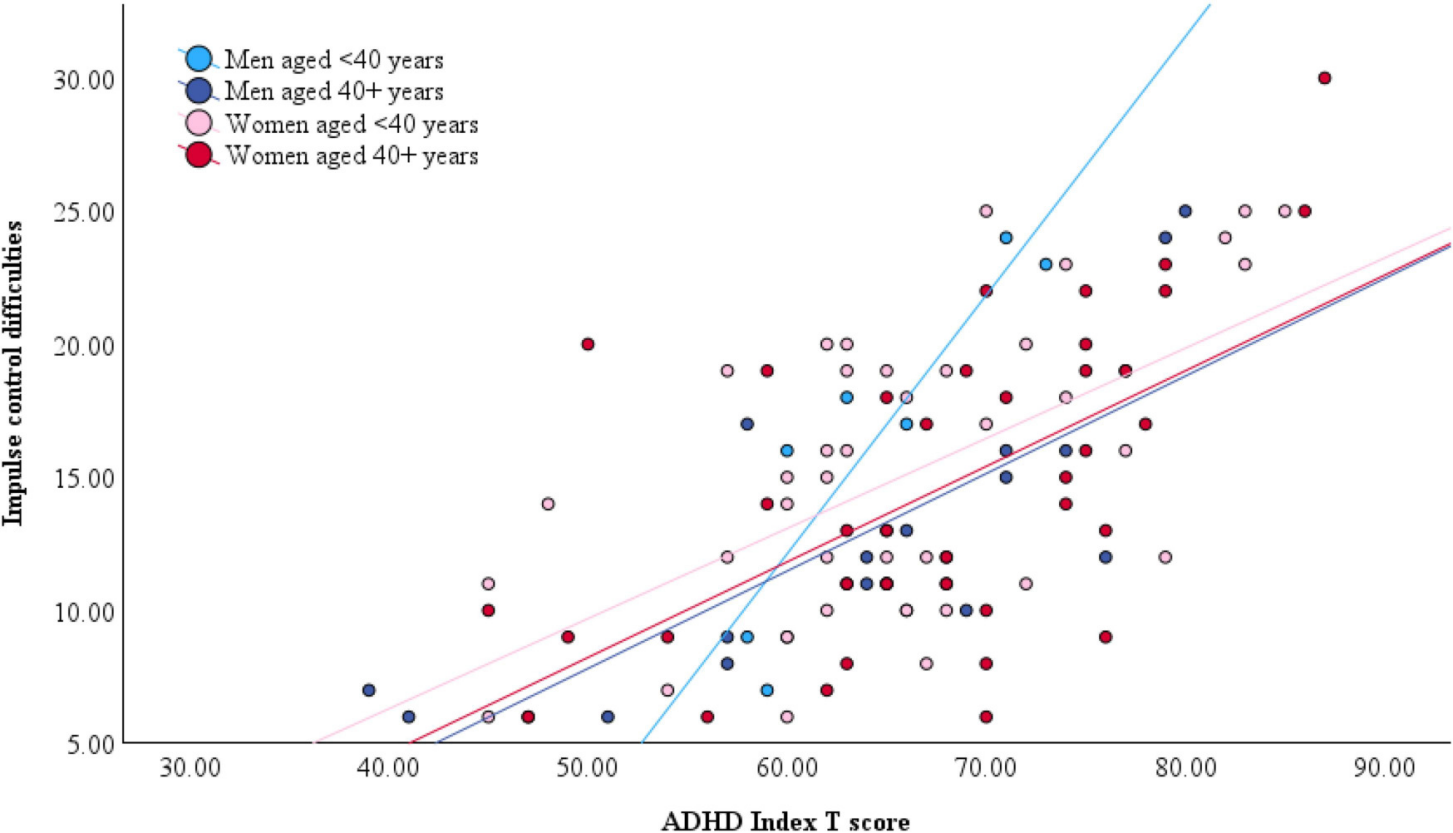
Gender-stratified relationship between impulse control difficulties, ADHD symptom severity, and age. Note that age was dichotomized by median split for illustrative purposes only. All models included age as a continuous predictor.

Age significantly moderated the relationship between ADHD symptoms and DERS impulse control difficulties in men, whereby the association was stronger in younger than in older men (*B* = −0.017, *p* = .012, *η*_p_^2^ = .28) (Figure 3). This association survived adjustments for depression and anxiety symptoms and the magnitude of the effect remained similar. There was no evidence of age moderation on any of the other emotional outcomes in women or men. Adjusted model parameters are summarized in Table 5.

**Table 4:** Gender-stratified association between ADHD symptoms, cognitive complaints, and age.

Women (*n* = 92)	*B*	*SE*	*t*	*p*	95% CI	*η*_p_^2^
Total CFQ						
Intercept	−43.962	24.332	−1.807	.074	[−92.332, 4.408]	.037
Age	0.882	0.521	1.691	.094	[−0.155, 1.919]	.032
ADHD index	1.805	0.364	4.958	<.001	[1.081, 2.528]	.222
Age * ADHD Index	−0.018	0.008	−2.223	.029	[−0.033, −0.002]	.054
BDEFS summary score						
Intercept	−1.363	16.843	−0.081	.936	[−34.847, 32.121]	.000
Age	−0.020	0.361	−0.056	.955	[−0.738, 0.697]	.000
ADHD index	0.841	0.252	3.339	.001	[0.340, 1.342]	.115
Age * ADHD Index	−0.002	0.005	−0.288	.774	[−0.012, 0.009]	.001
Time management						
Intercept	−0.863	5.149	−0.168	.867	[−11.098, 9.372]	.000
Age	0.072	0.110	0.656	.514	[−0.147, 0.292]	.005
ADHD index	0.228	0.077	2.955	.004	[0.074, 0.381]	.092
Age * ADHD Index	−0.001	0.002	−0.888	.377	[−0.005, 0.002]	.009
Organization						
Intercept	−1.653	6.816	−0.243	.809	[−15.203, 11.897]	.001
Age	0.053	0.146	0.361	.719	[−0.238, 0.343]	.002
ADHD index	0.170	0.102	1.670	.099	[−0.032, 0.373]	.031
Age * ADHD Index	−0.001	0.002	−0.385	.701	[−0.005, 0.004]	.002
Self-restraint						
Intercept	−1.290	5.235	−0.247	.806	[−11.697, 9.116]	.001
Age	−0.075	0.112	−0.669	.506	[−0.298, 0.148]	.005
ADHD index	0.166	0.078	2.122	.037	[0.011, 0.322]	.050
Age * ADHD index	0.001	0.002	0.442	.659	[−0.003, 0.004]	.002
Self-motivation						
Intercept	0.320	5.846	0.055	.957	[−11.301, 11.941]	.000
Age	−0.042	0.125	−0.337	.737	[−0.291, 0.207]	.001
ADHD index	0.163	0.087	1.863	.066	[−0.011, 0.337]	.039
Age * ADHD Index	0.000	0.002	−0.105	.916	[−0.004, 0.004]	.000
Self-regulation						
Intercept	2.124	4.476	0.475	.636	[−6.775, 11.023]	.003
Age	−0.028	0.096	−0.294	.769	[−0.219, 0.162]	.001
ADHD Index	0.114	0.067	1.708	.091	[−0.019, 0.247]	.033
Age * ADHD Index	0.000	0.001	0.144	.885	[−0.003, 0.003]	.000

Men (*n* = 25)	*B*	Std. Error	*t*	*p*	95% CI	*η*_p_^2^
Total CFQ						
Intercept	−88.905	66.427	−1.338	.197	[−227.937, 50.128]	.086
Age	0.926	1.087	0.852	.405	[−1.349, 3.200]	.037
ADHD index	2.397	1.019	2.352	.030	[0.264, 4.531]	.225
Age * ADHD Index	−0.018	0.017	−1.071	.297	[−0.054, 0.017]	.057
BDEFS summary score						
Intercept	59.487	47.860	1.243	.229	[−40.685, 159.658]	.075
Age	−0.789	0.783	−1.008	.326	[−2.428, 0.850]	.051
ADHD index	0.079	0.734	0.108	.915	[−1.458, 1.616]	.001
Age * ADHD Index	0.008	0.012	0.671	.510	[−0.017, 0.034]	.023
Time management						
Intercept	26.145	14.568	1.795	.089	[−4.346, 56.636]	.145
Age	−0.384	0.238	−1.611	.124	[−0.883, 0.115]	.120
ADHD index	−0.142	0.224	−0.634	.534	[−0.609, 0.326]	.021
Age * ADHD Index	0.005	0.004	1.287	.214	[−0.003, 0.013]	.080
Organization						
Intercept	24.969	20.315	1.229	.234	[−17.551, 67.488]	.074
Age	−0.338	0.332	−1.016	.322	[−1.033, 0.358]	.052
ADHD index	−0.201	0.312	−0.645	.527	[−0.854, 0.451]	.021
Age * ADHD Index	0.005	0.005	1.046	.308	[−0.005, 0.016]	.054
Self-restraint						
Intercept	−36.452	13.783	−2.645	.016	[−65.300, −7.604]	.269
Age	0.516	0.225	2.290	.034	[0.044, 0.988]	.216
ADHD index	0.708	0.211	3.348	.003	[0.266, 1.151]	.371
Age * ADHD Index	−0.009	0.004	−2.526	.021	[−0.016, −0.002]	.251
Self-motivation						
Intercept	27.408	18.303	1.497	.151	[−10.901, 65.717]	.106
Age	−0.338	0.299	−1.127	.274	[−0.964, 0.289]	.063
ADHD index	−0.191	0.281	−0.681	.504	[−0.779, 0.396]	.024
Age * ADHD Index	0.004	0.005	0.857	.402	[−0.006, 0.014]	.037
Self-regulation						
Intercept	17.417	14.706	1.184	.251	[−13.362, 48.197]	.069
Age	−0.246	0.241	−1.023	.319	[−0.750, 0.257]	.052
ADHD index	−0.095	0.226	−0.421	.679	[−0.567, 0.377]	.009
Age * ADHD Index	0.003	0.004	0.766	.453	[−0.005, 0.011]	.030

Parameters shown are adjusted for symptoms of depression and anxiety. Effect sizes are shown as partial eta squared (η_p_^2^) where .01 indicates a small effect,.06 indicates a medium effect and .14 indicates a large effect. ADHD, attention-deficit/hyperactivity disorder; CFQ, Cognitive Failures Questionnaire; BDEFS, barkley deficits in executive functioning scale; SE, standard error.

**Table 5:** Gender-stratified association between age and emotional dysregulation.

Women (*n* = 92)	*B*	*SE*	*t*	*p*	95% CI	η_p_^2^
DERS total						
Intercept	20.432	36.459	0.560	.577	[−52.138, 93.001]	.004
Age	−0.298	0.774	−0.385	.701	[−1.839, 1.243]	.002
ADHD index	1.008	0.544	1.852	.068	[−0.075, 2.091]	.042
Age * ADHD Index	0.002	0.012	0.189	.851	[−0.021, 0.026]	.000
Nonacceptance						
Intercept	7.243	14.053	0.515	.608	[−20.728, 35.214]	.003
Age	−0.118	0.298	−0.396	.693	[−0.712, 0.476]	.002
ADHD index	0.190	0.210	0.904	.369	[−0.228, 0.607]	.010
Age * ADHD Index	0.001	0.005	0.306	.761	[−0.008, 0.010]	.001
Goal-directed behavior						
Intercept	4.184	7.967	0.525	.601	[−11.674, 20.043]	.003
Age	−0.014	0.169	−0.084	.933	[−0.351, 0.323]	.000
ADHD index	0.285	0.119	2.400	.019	[0.049, 0.522]	.068
Age * ADHD Index	−0.001	0.003	−0.238	.813	[−0.006, 0.005]	.001
Impulse control						
Intercept	−0.222	9.971	−0.022	.982	[−20.069, 19.626]	.000
Age	−0.145	0.212	−0.683	.496	[−0.566, 0.277]	.006
ADHD index	0.266	0.149	1.790	.077	[−0.030, 0.563]	.039
Age * ADHD Index	0.002	0.003	0.499	.619	[−0.005, 0.008]	.003
Emotional awareness						
Intercept	10.276	11.537	0.891	.376	[−12.689, 33.241]	.010
Age	0.042	0.245	0.172	.864	[−0.446, 0.530]	.000
ADHD index	0.069	0.172	0.402	.689	[−0.274, 0.412]	.002
Age * ADHD Index	0.000	0.004	0.113	.911	[−0.007, 0.008]	.000
Emotion regulation strategies						
Intercept	10.767	13.145	0.819	.415	[−15.399, 36.932]	.008
Age	−0.058	0.279	−0.207	.836	[−0.613, 0.498]	.001
ADHD index	0.275	0.196	1.400	.165	[−0.116, 0.665]	.024
Age * ADHD Index	0.000	0.004	0.113	.910	[−0.008, 0.009]	.000
Emotional clarity						
Intercept	5.608	9.651	0.581	.563	[−13.601, 24.817]	.004
Age	−0.052	0.205	−0.255	.800	[−0.460, 0.356]	.001
ADHD index	0.124	0.144	0.860	.392	[−0.163, 0.411]	.009
Age * ADHD Index	0.001	0.003	0.262	.794	[−0.005, 0.007]	.001

Men (*n* = 25)	*B*	Std. Error	*t*	*p*	95% CI	η_p_^2^
DERS total						
Intercept	13.124	70.077	0.187	.854	[−134.102, 160.351]	.002
Age	−0.512	1.100	−0.465	.647	[−2.824, 1.800]	.012
ADHD index	1.185	1.082	1.095	.288	[−1.089, 3.459]	.062
Age * ADHD Index	0.002	0.018	0.139	.891	[−0.034, 0.039]	.001
Non-acceptance						
Intercept	48.064	41.448	1.160	.261	[−39.015, 135.143]	.070
Age	−0.712	0.651	−1.093	.289	[−2.079, 0.656]	.062
ADHD index	−0.405	0.640	−0.633	.534	[−1.750, 0.940]	.022
Age * ADHD Index	0.011	0.010	1.014	.324	[−0.011, 0.032]	.054
Goal-directed behavior						
Intercept	37.161	29.206	1.272	.219	[−24.200, 98.521]	.083
Age	−0.560	0.459	−1.220	.238	[−1.523, 0.404]	.076
ADHD Index	−0.263	0.451	−0.582	.568	[−1.210, 0.685]	.018
Age * ADHD Index	0.008	0.007	1.145	.267	[−0.007, 0.024]	.068
Impulse control						
Intercept	−67.820	24.070	−2.818	.011	[−118.388, −17.252]	.306
Age	0.905	0.378	2.394	.028	[0.111, 1.699]	.241
ADHD index	1.336	0.372	3.593	.002	[0.555, 2.117]	.418
Age * ADHD Index	−0.016	0.006	−2.595	.018	[−0.028, −0.003]	.272
Emotional awareness						
Intercept	26.865	37.559	0.715	.484	[−52.045, 105.774]	.028
Age	−0.432	0.590	−0.732	.473	[−1.671, 0.807]	.029
ADHD index	−0.158	0.580	−0.272	.789	[−1.377, 1.061]	.004
Age * ADHD Index	0.006	0.009	0.666	.514	[−0.013, 0.026]	.024
Emotion regulation strategies						
Intercept	5.421	30.298	0.179	.860	[−58.233, 69.074]	.002
Age	−0.285	0.476	−0.598	.557	[−1.284, 0.715]	.019
ADHD index	0.379	0.468	0.810	.429	[−0.604, 1.362]	.035
Age * ADHD Index	0.002	0.008	0.299	.768	[−0.014, 0.018]	.005
Emotional clarity						
Intercept	6.888	16.994	0.405	.690	[−28.815, 42.591]	.009
Age	−0.214	0.267	−0.803	.432	[−0.775, 0.346]	.035
ADHD index	0.111	0.262	0.424	.677	[−0.440, 0.663]	.010
Age * ADHD index	0.002	0.004	0.389	.702	[−0.007, 0.011]	.008

Parameters shown are adjusted for symptoms of depression and anxiety. Effect sizes are shown as partial eta squared (η_p_^2^) where 0.01 indicates a small effect, 0.06 indicates a medium effect and 0.14 indicates a large effect. ADHD, attention-deficit/hyperactivity disorder; DERS, difficulties in emotion regulation scale; SE, standard error.

## 4 Discussion

Does age impact the subjective cognitive and emotional difficulties associated with ADHD symptoms differently in men compared to women? To answer this question, the present study conducted gender-stratified tests of moderating age effects in a cross-sectional sample of adults 19–79 years old. Due to age-related hormonal changes, older women were expected to experience worse ADHD-related cognitive and emotional difficulties relative to younger women and to men. Results showed, first, that ADHD symptoms were moderately predictive of subjective cognitive and emotional difficulties in both men and women. Second, gender differences were indeed observed in the moderating effect of age on some ADHD-related outcomes, but these findings were generally driven by age-related *decreases* in men's self-reported difficulties (rather than age-related *increases* in women's difficulties, contrary to our hypothesis). In contrast, women's self-reported cognitive and emotional difficulties were comparable across different ages. These findings are interpreted below.

### 4.1 Men’s ADHD-related impulsivity may improve with age

In men, age significantly moderated the impact of ADHD symptoms on two measures, both of which index impulse control. The BDEFS Self-Restraint subscale refers to one's inability to inhibit reactions or responses, the tendency to make impulsive comments to others, and to act without thinking. The DERS Impulse Control Difficulties subscale describes feeling out of control when upset and being unable to control overwhelming emotional experiences. Thus, both these subscales refer to self-regulatory control of behavior or emotion, processes which are subserved by the prefrontal cortex (68). Although men and women showed comparable mean scores for both measures, the moderating effect of age was only evidence in men: the association between ADHD symptoms and impulse control was weaker in older men than in younger men. Effect sizes were large for both BDEFS and DERS indices of impulse control. In other words, for a given level of ADHD symptoms, older men reported much fewer impulse control difficulties relative to younger men, but this was not true for women.

It is possible to tentatively infer from these results that impulsivity may improve with age in men with ADHD, a finding which would be consistent with other reports of age-related decreases in some forms of impulse control in neurotypical men [e.g., (69)]. Although this can only be confirmed in longitudinal investigations, the possibility of age-related impulse control improvements in ADHD may have optimistic implications for aging in men with ADHD. Impulsivity is associated with many adverse outcomes in ADHD, including increased substance abuse problems and risky sensation seeking (70) as well as occupational, criminal, driving, and financial difficulties (71), and improvements in self-regulatory processes may indicate better outcomes in these domains in later life. Long-term follow-up of men with ADHD would be valuable to ascertain the extent and impact of possible changes in impulsivity.

### 4.2 Age does not modify the subjective cognitive and emotional difficulties associated with ADHD symptoms

The picture emerging from the data in the present study is one of stability in women's subjective cognitive and emotional complaints related to ADHD symptoms across age groups. Women of different ages in this sample reported generally similar levels of difficulties, regardless of their ADHD symptom severity. The exception to this pattern was a small- to medium-sized age-related decrease in the association between ADHD symptoms and total CFQ score in women. The CFQ asks about everyday cognitive mistakes related to distractibility, forgetfulness, inattention, and word slips. These cognitive concerns were reported frequently—of a total possible score of 100, women's average score was 60—and were moderately correlated with ADHD symptom severity. Rather surprisingly, CFQ scores were inversely correlated with age for both genders [despite known cognitive declines in normal aging: (72)], and age further moderated the association between ADHD symptoms and CFQ, whereby older women reported slightly but significantly fewer cognitive complaints than younger women with an equivalent level of ADHD symptoms. In men, this trend was also apparent and of comparable moderate magnitude but did not reach statistical significance, likely due to sample size. Previously, there have been other published reports of older adults reporting fewer cognitive difficulties on the CFQ relative to younger adults (73–75). As explanations for these counterintuitive findings, authors have suggested the possibility that older adults may actually experience fewer everyday cognitive slips as a result of reduced demands following retirement and lifestyle changes, or alternatively that they do experience cognitive difficulties but are inaccurate in monitoring and reporting them (73). In the present sample, another possibility could be that older participants have already been experiencing ADHD-related cognitive slips for many decades, and do not identify them as ‘complaints’ but rather as how their brain has always functioned. It is impossible for the present study to adjudicate this question without any objective cognitive measures or informant reports to use as ancillary evidence. However, this question is one that deserves further investigation because it has implications for how age-related cognitive processes are conceptualized in ADHD and the extent to which we can rely on subjective reports of difficulty in aging.

Women's cognitive and emotional challenges linked to ADHD symptoms were otherwise stable across different age groups in the present study. At the outset, it was expected that self-reported difficulties would be worse in older women relative to younger women and to men. This hypothesis was based on known mid-life changes in cognitive and emotional health that arise concurrent to menopause (33, 34), and it was anticipated that existing ADHD-related difficulties would be exacerbated by menopausal reductions in estrogen and dopamine levels (40). The fact that cognitive or emotional challenges were not markedly worse in older women in this sample is relatively more consistent with descriptions of potential menopausal changes as being isolated to the perimenopausal period, rather than persistent and lasting (32). Yet, this finding is in direct contradiction to results from a reader survey of more than 1,500 women conducted by ADDitude Magazine, in which 94% of respondents reported experiencing the most severe ADHD-related impairments of their life during perimenopause and menopause (76). Clearly, much remains to be understood about women's age-related changes in ADHD symptoms and associated impairments linked to menopause, and there is a critical need for further general research about links between ADHD and sex hormones in girls and women.

### 4.3 Implications for understanding aging with ADHD

ADHD symptoms were broadly associated with subjective cognitive and emotional complaints. The lack of any observed age-related increases in cognitive complaints is reassuring from a dementia risk perspective, but the robustness of this finding will need to be replicated in larger samples. It would also be valuable for future work to use objective measures of cognitive processes alongside subjective estimates, as evidence from younger samples suggests that both types of data provide complementary information about cognitive performance (77). It will be useful to establish the validity of this phenomenon in older cohorts.

ADHD symptoms were also significantly associated with participants’ self-reports of nonacceptance of emotional responses, difficulties engaging in goal-directed behavior when upset, difficulties controlling emotional impulses, lack of emotional awareness, difficulties using emotion-regulation strategies, and lack of emotional clarity. None of these challenges—with the exception of emotional impulse control for men—showed any meaningful age-related change in this sample. We may cautiously interpret from this finding the possibility that emotional processes in adults with ADHD do not follow the age-related improvements predicted by socioemotional selectivity theory (27). Indeed, qualitative interviews of adults aged 50+ with ADHD highlight peer rejection and family conflict as the most frequently mentioned challenges by participants (78, 79). Although these challenges do not necessarily imply emotional dysregulation *per se*, poor friendship and romantic relationship quality in adults with ADHD are significantly predicted by emotion regulation problems (80). Future studies seeking to confirm reduced socioemotional selectivity among older adults with ADHD should employ a range of tasks previously employed in the broader literature, including those that measure the construct implicitly.

### 4.4 Limitations and future directions

This evidence presented here should be considered preliminary in light of several methodological limitations that may be addressed in future work.

First, the cross-sectional design precludes any conclusions about causality and introduces the possibility that results may be driven by cohort bias wherein older men and women may self-report (or under-report) certain kinds of complaints because of generational influences unrelated to aging. While cross-sectional studies are valuable to uncover associations between variables of interest, they only capture a snapshot of the data at one time point and do not provide information about the directionality of these associations. The time point at which data were collected is not necessarily an accurate reflection of the groups’ more general behaviors. Thus, the gender differences observed in this study will need to be tested in cohorts followed over time to robustly ascertain how ADHD-related cognitive and emotional complaints evolve longitudinally as individuals age.

Second, all outcome measures in the present study were self-reported, an approach deliberately taken to explore participants’ subjective difficulties. However, women may be slightly more likely to endorse ADHD-related impairment (81), and it is possible that age-related “decreases” in cognitive and emotional impulsivity in men actually reflect lower self-disclosure in older men relative to women. It is also possible that people of different ages may have different biases in how they perceive their own symptoms. Older adults, for example, may downplay their symptoms as a result of an age-related positivity bias (26) or because of memory changes that compromise their retrospective estimates of symptoms or behaviors (6). Data collected from informants (e.g., family members) may have provided different results from those observed in the present study.

Third, various clinical presentations unrelated to ADHD can include features of inattention, hyperactivity and impulsivity, as well as dysfunction in memory, executive abilities and emotional regulation. These features are nonspecific to ADHD and it is therefore possible that pathological processes unrelated to ADHD may have driven some of the results reported here. Even so, we estimate that this is not likely to be a significant threat to the findings given that most participants reported having received a formal diagnosis of ADHD from their healthcare provider (71%) or scored above the ASRS clinical threshold (78%) that is 94% specific to ADHD. Nonetheless, the ASRS alone cannot be used to confirm an ADHD diagnosis, and we cannot definitively confirm that Canadian ADHD Practice Guidelines were rigorously applied in all cases where a formal diagnosis was self-reported. Future work should seek to confirm the observed associations in a sample with confirmed ADHD diagnosis.

Finally, results are interpreted cautiously in the context of relatively small sample size, particularly with regard to male participants. This sample was underpowered to explore age effects across specific developmental stages, but future work in this regard would provide a richer, more nuanced perspective on ADHD outcomes across the lifespan.

### 4.5 Conclusion

Some cognitive and emotional difficulties associated with ADHD symptoms showed age-related declines in men but not women, tentatively suggesting that women with ADHD may experience greater challenges as they age relative to men with ADHD. Altogether, results support the need for a gender-specific lens when considering the lifespan impacts of ADHD symptoms and potentially point to women with ADHD as an especially vulnerable segment of the population regarding cognitive and emotional health.

## Data Availability

The raw data supporting the conclusions of this article will be made available by the authors, without undue reservation.
